# Characterization of the Key Aroma Compounds in Chinese Syrah Wine by Gas Chromatography-Olfactometry-Mass Spectrometry and Aroma Reconstitution Studies

**DOI:** 10.3390/molecules22071045

**Published:** 2017-06-24

**Authors:** Pengtao Zhao, Jinxin Gao, Michael Qian, Hua Li

**Affiliations:** 1 College of Enology, Northwest A&F University, Yangling 712100, Shaanxi, China; zpt5232@Gmail.com (P.Z.); jasminexy2016@icloud.com (J.G.); 2 Department of Food Science & Technology, Oregon State University, Corvallis, OR 97333, USA; Michael.qian@oregonstate.edu; 3 Shaanxi Engineering Research Center for Viti-Viniculture, Yangling 712100, Shaanxi, China

**Keywords:** Syrah wine, aroma compounds, GC-O, SBSE, OAV, sensory descriptive analysis

## Abstract

The key aroma compounds and the organoleptic quality of two Chinese Syrah wines from the Yunnan Shangri-La region and Ningxia Helan mountain region were characterized. The most important eighty aroma-active compounds were identified by Gas Chromatography-Olfactometry. In both Syrah samples, ethyl 2-methylpropanoate, ethyl 3-methylbutanoate, 3-methylbutyl acetate, 2- and 3-methyl-1-butanol, ethyl hexanoate, ethyl octanoate, 2-phenethyl acetate, methional, 3-methylbutanoic acid, hexanoic acid, octanoic acid, *β*-damascenone, guaiacol, 2-phenylethanol, *trans*-whiskylactone, 4-ethylguaiacol, eugenol, 4-ethylphenol, and sotolon were detected to have the highest odor intensities. In the chemical analysis, 72 compounds were quantitated by Stir Bar Sorptive Extraction combined with Gas Chromatography Mass Spectrometry. Based on the Odor Activity Value (OAV), the aromas were reconstituted by combining aroma compounds in the synthetic wine, and sensory descriptive analysis was used to verify the chemical data. Fatty acid ethyl esters, acetate esters, and *β*-damascenone were found with higher OAVs in the more fruity-smelling sample of Helan Mountain rather than Shangri-La.

## 1. Introduction

Wine aroma characteristics originate from grape berries, wine fermentation, and aging techniques [1,2]. Over one thousand volatile and semi-volatile compounds have been identified in wine contributing to its aroma quality [3]. High contributing aroma-active volatiles in wine include alcohols, esters, fatty acids, ketones, terpenes, phenols, and aldehydes. They exist in varying concentrations, from 10^−12^ to 10^−4^ g/L [4,5]. Volatiles in wine are complex and heterogeneous, and those concentrations that are higher than their odor threshold are considered as potential contributors to wine aroma [6,7,8,9].

Solid Phase Extraction (SPE) based on LiChrolut-EN is a fast and user friendly technique that has been widely utilized to isolate and concentrate volatile compounds in wine [10]. This technique concentrates and purifies the analytes by adsorbing the compounds onto the resin bed and recovering them using a small volume of suitable solvents [11]. Solid Phase Microextraction (SPME) and Stir Bar Sorptive Extraction (SBSE) techniques based on polydimethylsiloxane (PDMS) as the absorbent for nonpolar and intermediate polar compounds are of high selectivity and only require a small amount of sample. They are rapid and easy to perform techniques, and their use has been increasing for the analysis of different types of volatile compounds in wine [9,10,11,12]. Compared to SPME, SBSE is far more sensitive since its stir bar has much more sorbent volume to extract and concentrate analytes from a larger sample volume [13]. SBSE could be performed for the extraction of those compounds with low concentration or low affinity. PDMS coated stir bars and ethylene glycol-silicone (EG) coated stir bars are the two most frequently used extraction materials in wine flavor chemistry research [12,13,14]. Nie and Albinus [15] reported that both EG-Silicone-SBSE and derivatization-PDMS-SBSE types were successfully applied for the quantitative analysis of volatile phenols in beverages. Good linearity and repeatability were obtained.

With the application of gas chromatography-olfactometry (GC-O), the most potent odorants in wine have been identified. Meanwhile, by quantitative analysis of the aroma-active compounds, odor activity values (OAVs) of the potential contributors were obtained, which reflect their significance to the aroma profile of wine. The connection of flavor chemistry with GC-O techniques has allowed for associating wine sensory properties to specific chemical compounds [16,17,18,19,20].

Syrah (*Vitis vinifera* L. cv.) originally from Rhone valley (France) has been very popular for its full-bodied, dark fruit flavors, berry-like aroma, and is often described as having spicy or pepper-like attributes [21]. Despite the importance of Syrah to the wine industry, little is known about the aroma compounds that are responsible for the perceived aroma of Syrah wine. Wine grapes grown in different climatic and geographical areas led to a diverse expression of varietal characteristics [22]. Mayr et al. [23] characterized two premium Syrah wine from Australia using GC-O, quantitation, and aroma reconstitution techniques. Approximately 60 odorants were detected from the liquid extract and the dynamic headspace of Syrah wine; ethyl butanoate, ethyl 2-methylbutanoate, ethyl 3-methylbutanoate, 2- and 3-methyl-1-butanol, ethyl hexanoate, furfuryl ethyl ether, *β*-damascenone, guaiacol, and 2-phenylethanol were detected to have the highest Aroma Extract Dilution Analysis (AEDA) values. They selected the 48 most important compounds that were quantitatively analyzed. According to OAV, ethyl octanoate, ethyl hexanoate, ethyl 3-methylbutanoate, ethyl 2-methylbutanoate, ethyl acetate, *β*-damascenone, 3-methyl butanoic acid, eugenol, and *cis*-whisky lactone were found to be the most significant contributors to Syrah wine aroma.

With the rapid development of the wine industry in China, the quality of Chinese wine is improving quickly and appeals to more and more consumers. The Shangri-La plateau region located in the core area of Yunnan province, southwestern China, is one of the world's highest altitude vineyards with an average elevation of over 2400 m. The highest quality vineyard on both sides of the Jinsha River, Lancang River valley slope is one of the most characteristic vineyards in China [24]. The Ningxia eastern Helan Mountain region located in the northwestern region of China is a wide, heavily irrigated valley between the Yellow River and the base of Helan Mountain. Ningxia has a thoroughly continental climate and high altitude of the vineyards (more than 1000 m above sea level). It has proved to be one of China’s most promising vineyard areas [25].

In the past few decades, wine from China, especially Helan Mountain and Shangri-La, are attracting attention from all over the world and earning more and more recognition. Winemakers are therefore further investing in their potential. However, no comprehensive aroma characterization of Syrah wine in China has been conducted. The present paper aimed to determine the key aroma and aroma potentials of two Syrah wines from China. The main correlations between the chemical composition and the sensory properties were established.

## 2. Results

### 2.1. Identification of Aroma Compounds by GC-O

Potential volatile fractions in the concentrated extracts were determined by GC-O. The results listed in Table 1 show 79 compounds that were detected in the two Syrah wines, including esters, higher alcohols, volatile phenols, fatty acids, ketones and lactones, aromatic compounds, and sulfides, which were determined to play significant roles in wine aroma quality. In the GC-O study, the odorants in the extracts were described as fruity, floral, green, spicy, tobacco, woody, potato, nutty, herb, brandy, sweaty, and so on.

The highest intensities of the compounds identified in Ningxia Syrah sample (NS) and Yunnan Syrah sample (YS) were found for ethyl 2-methylpropanoate, ethyl 3-methylbutanoate, 3-methylbutyl acetate, 2- and 3-methyl-1-butanol, ethyl hexanoate, ethyl octanoate, 2-phenethyl acetate, methional, 3-methylbutanoic acid, hexanoic acid, octanoic acid, *β*-damascenone, guaiacol, 2-phenylethanol, *trans*-whisky lactone, 4-ethylguaiacol, eugenol, 4-ethylphenol, and sotolon. They were detected to have odor intensities ≥3.0 in at least one Syrah extract. The two Syrah wine samples showed very similar odorant contents and the proportion of volatile components. Esters (21 compounds), volatile phenols (13), fusel alcohols (11), fatty acids (8), and C_13_-norisoprenoids (2) groups were considered as the main contributors to wine aroma. The aroma contributors of Chinese Syrah reported in this study were consistent with the research by Mayr et al. [23] on two Syrah wine from the warmer Barossa Valley and the cooler Margaret River Syrah.

Some studies reported links of odorants with sensory attributes in wine which showed positive correlations between fruity (berry or dried fruit) and ethyl ester compounds [21,26], woody aromas, and volatile phenols [27]. Straight and branched chain fatty acids such as propionic acid, 3-methylbutyric acid, hexanoic acid, and octanoic acid in wine (10^−6^ g/L level concentration) were considered as unpleasant odorants, but they contribute significantly to the complexity of the wine total flavor [28]. C_13_-norisoprenoid compounds *β*-damascenone (fruity-flowery smelling) and *β*-ionone (violet smelling) were reported frequently in premium wine [29,30]. To understand the correlations between volatile compounds and aroma attributes, two samples are comparatively discussed in the GC-O section, quantitative analysis section, and aroma reconstitution test section. Compared with YS, NS showed higher intensities in odorants: 3-methylbutyl acetate, ethyl hexanoate, ethyl octanoate, methional, benzaldehyde, ethyl decanoate, guaiacol, *trans*-whiskylactone, *γ*-decalactone, sotolon, and ethyl vanillate. These compounds mainly contribute to the fruity, nutty, and clove characters of wine. In contrast, compounds 1,1-diethoxyethane, 2-methylpropanoic acid, α-terpineol, octanoic acid, and *m*-cresol in NS were detected to have lower odor intensities than that in YS.

### 2.2. Quantitative Analysis of Aroma-Active Compounds

To gain insight into the aroma characteristics of the two Syrah wines, 72 important compounds (shown in Table 2), from the GC-O data and from the literature [23,31] were quantitated and their odor activity values (OAVs) were calculated. As expected, volatiles were detected at levels ranging from ng/L to mg/L. The highest concentration in both wines was found for isoamyl alcohol (123 mg/L for NS and 106 mg/L for YS), followed by ethyl acetate, isobutyl alcohol, propanol, diethyl succinate, 2-phenethanol, and acetaldehyde. Since those fermentation byproducts have a relatively high odor threshold (especially diethyl succinate of 120 mg/L and 2-phenethanol of 14 mg/L), they were usually not necessarily considered as high aroma contributors to wine. Among the 72 quantitated compounds, in the Ningxia Syrah wine, 18 compounds reached concentrations higher than their odor threshold, whereas in the Shangri-La Syrah, 19 compounds were found to have an OAV > 1. Those aroma-active compounds (23 compounds in NS and 24 compounds in YS) with OAV between 0.1 and 1 may also contribute to the formation of wine aroma due to the interaction effect in the wine matrix. In the wine from the Ningxia Helan Mountain region, these fusel alcohols were at higher concentrations than that in YS.

According to the odor threshold, the highest OAV in the Syrah from the Ningxia region was determined for *β*-damascenone (tobacco and burnt sugar smelling, OAV 56.1), followed by the fruity-like ethyl octanoate (OAV 54.9), and acetaldehyde (fruity and musty or pungent smelling, OAV 44.7). The fruity straight and branched esters ethyl isovalerate (OAV 43.8), ethyl hexanoate (OAV 38.1), ethyl isobutyrate (OAV 17.6), and isoamyl acetate (OAV 13.6) also had high OAVs are therefore very significant aroma contributors in NS. Those ester compounds produced by yeasts during fermentation are well known to contribute to and enhance the fruity aroma of wines [32]. *β*-Damascenone and *β*-ionone mainly come from the degradation of carotenoids in grapes, and are well known as important characteristic flavor compounds in wine, which were characterized by “fruity-flowery” and “violet” notes [33,34]. They are important C_13_-norisoprenoids due to their low odor threshold and high contribution to wine aroma complexity [29,30]. Fatty acids are the precursors of esters, terpenes, and alcohols in grapes and wine that mainly contribute to the flavor of wine [32]. Butanoic acid, 2- and 3-methylbutyric acid, hexanoic acid, and octanoic acid that give a cheesy or sweaty smell and contribute complexity to wine also had concentrations higher than their thresholds. Volatile acids in wine are important components in flavor quality; they impart woody, brandy, almond, etc., pleasant aromas to wine in proper concentration levels, while high concentrations (above 20 mg/L) of acids will give a negative aroma hints of wine [20,21]. Oak-derived phenolic compounds guaiacol (OAV 1.25), eugenol (OAV 1.12) and ethyl dihydrocinnamate (OAV 1.05) were also found in concentrations above their thresholds, and they were reported as important aroma potentials in aged wine. Most of these compounds are formed through hydrolysis of glycosidically conjugated forms during wine aging, or are extracted from oak during barrel aging, contributing floral and cherry notes to wine [3,4,5,35].

In the Syrah wine from the Shangri-La region, compounds with the highest OAVs were mostly similar to those in NS. Unlike Syrah from the Ningxia region, acetaldehyde (OAV 63.8) was the highest in YS. The fruity-smelling ester compounds ethyl isovalerate (OAV 27.3), ethyl octanoate (OAV 11.7), ethyl hexanoate (OAV 10.8), ethyl isobutyrate (OAV 10.9), and isoamyl acetate (OAV 8.1) in YS were detected to have much lower OAVs compared with those in NS. The high concentration of acetaldehyde and lower concentration of esters in YS were supposed to be generated during relatively high alcohol fermentation temperatures. According to the record profile of the winery, the atmospheric temperature during the alcohol fermentation was higher than usual without a cooling system. Molina et al. [36] and Culleré et al. [37] reported that higher wine fermentation temperature interferes with the reactions of esters and increases the formation of acetaldehyde. Besides, the addition of SO_2_ before alcohol fermentation may affect the formation of acetaldehyde in wine. The Syrah wine from the Shangri-La region in this study was determined to have a lower concentration of esters and lower intensities of fruity odors. Mayr et al. [23] determined two Australian Syrah wines. The wine from the warmer Barossa Valley was found to have higher concentrations of ethyl propionate and oak-derived compounds, whereas the cooler Margaret River Syrah had above threshold concentrations of 2- and 3-methylbutanoic acid, as well as rotundone. *β*-Damascenone (OAV 24.4) was found with a lower odor activity value in YS than NS, as well as the fatty acids 2- and 3-methylbutyric acid (OAV 11.8).

The oak-derived compounds guaiacol (OAV 1.58) and eugenol (OAV 1.19) were detected to have similar concentrations in two Syrah wines. Compared with those in NS, ethyl acetate (OAV 6.62), *trans*-whiskylactone (OAV 1.82), vanillin (OAV 0.5–1), and phenolic compounds (all with OAV 0.5–1): 4-methylguaiacol, 4-ethylguaiacol, isoeugenol, 4-ethylphenol, and 3-ethylphenol were found to have higher odor activity values in YS. Fusel alcohols including propanol, isobutanol, 2- and 3-methyl-1-butanol, 1-hexanol, benzyl alcohol, and 2-phenethanol in wine are mainly formed through alcoholic fermentation from sugar and amino acid catabolism. They might show either positive or negative impacts on total aroma depending on the concentration level. It was reported that a level of higher alcohols below 300 mg/L confers a desirable complexity to the wine whereas concentrations over 400 mg/L have a negative effect [38]. In this study, both wines had fusel alcohols below 300 mg/L, which contributed positively to the wine quality. 2-Phenylethanol that gave wine a positive rose aroma was also reported as a significant compound. In NS and YS wine, linalool and rose oxide have OAV between 0.1 and 0.5; those terpenoids are responsible for the odor of citrus (lemon) and floral aromas in wine. Furans and lactones with low odor thresholds in wine were always reported as the typical compounds in the aging wine. In this study, the low OAV of this group of compounds was consistent with the young wine vintage. Methoxypyrazine compounds 3-isopropyl-2-methoxypyrazine (IPMP), 2-*sec*-butyl-3-methoxypyrazine (SBMP), and 3-isobutyl-2-methoxypyrazine (IBMP) contributing green bell pepper or bean notes to wine had very low odor thresholds (IBMP, 2 ng/L). The concentrations of methoxypyrazines in the sample wines did not reach the threshold, but they might be potential important odorants in wine due to the synergistic effect of the interaction between compounds [7,39].

### 2.3. Sensory Evaluation of Syrah Wine Samples and Aroma Reconstitution Test

**Figure 1:**
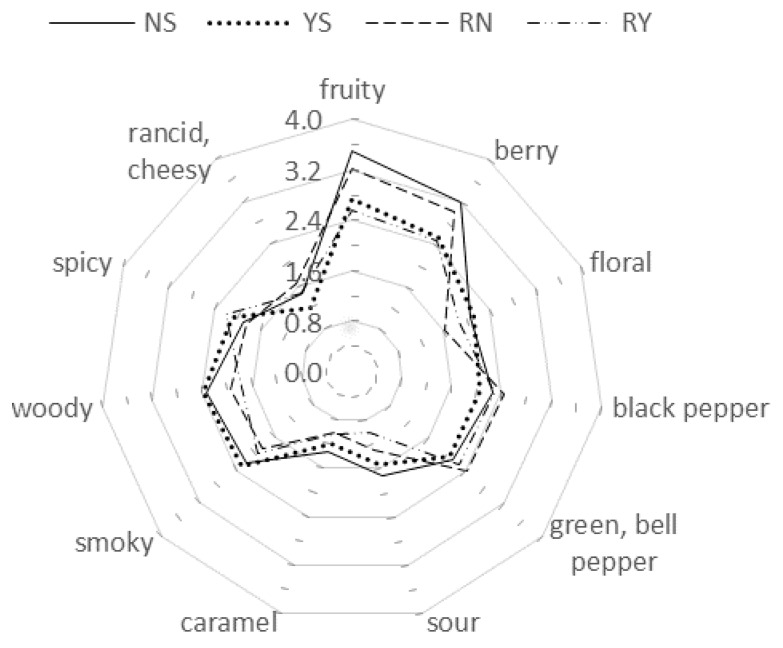
Aroma profiles of Syrah wine samples and reconstitution wine. Symbols in the figure: NS (Ningxia Syrah), YS (Yunnan Syrah), RN (Reconstitution NS), RY (Reconstitution YS).

Sensory descriptive analysis was performed to compare the aroma differences of the Syrah wine samples NS and YS, and to relate aroma compounds with sensory attributes. As shown in Figure 1, the two Syrah wines had similar intensities of floral, black pepper, green or bell pepper, sour, caramel, smoky, woody, spicy, and rancid or cheesy attributes. NS had a higher score of fruity (3.5) and berry (3.2) terms (*p* < 0.05) than that of YS (2.7 and 2.5, respectively), which could be explained by the concentration differences of the esters with high OAVs and their synergetic interactions. For other attributes, the two wines showed similar results: the panelists could not detect the difference. Figure 1 illustrates that fruity, berry, floral, sour, caramel, smoky, and woody terms showed lower intensities in reconstitution wine than that in the wine samples.

## 3. Materials and Methods

### 3.1. Wines

Two popular commercial Syrah wines of vintage 2014 were kindly donated by wineries. NS was from the Ningxia region in northwest China. The basic composition of the wine was as follows: alcohol 14.1% *v*/*v*, pH 3.48, titratable acidity (TA, as tartaric acid) 6.78 g/L, volatile acidity (VA, as acetic acid) 0.78 g/L, total SO_2_ 64 mg/L, glucose + fructose (G + F) 1.2 g/L. YS was from a high altitude region, Yunnan Shangri-La in southwest China. The basic compositions: alcohol percentage of 13.6% by volume, pH 3.56, TA 6.51 g/L, VA 0.51g/L, total SO_2_ 91 mg/L, G + F 2.0 g/L. Both wine samples were fermented in stainless steel fermenters at 20–32 °C, and the malolactic fermentation was performed ten days after the alcohol fermentation. Both wines were aged in the same type of France oak for half a year and bottled. Once received, the wine samples were stored in a 4 °C controlled temperature room.

### 3.2. Reagents and Chemical Standards

Chemical standards of the compounds in this study were purchased from Sigma-Aldrich (St. Louis, MO, USA), TCI America (Portland, OR, USA), EKC Inc. (Rosemont, IL, USA), Alfa Aesar (Ward Hill, MA, USA), and EMD Chemical Inc. (Gibbstown, NJ, USA), and their purities were >90% in all cases. The details are shown in a supplementary table. Milli-Q quality water was obtained from a Milli-Q purification system (Millipore, Boston, MA, USA). Methanol (HPLC grade) was from EM Science (Gibbstown, NJ, USA). Acetonitrile (HPLC grade) was bought from Omnisolv (McLean, VA, USA). Dichloromethane (HPLC grade) from Burdick & Jackson (Muskegon, MI, USA) was freshly distilled before use. Tartaric acid was from Mallinckrodt Inc. (Paris, France). Anhydrous sodium sulfate and sodium chloride (99.9%, ACS certified) were supplied by Mallinckrodt Baker. The LiChrolut-EN cartridge with a two-gram absorbent bed was packed in the laboratory.

Standard stock solutions were prepared in methanol individually except that fatty acids were in acetonitrile. Internal standard solution (IS) of 3,4-dimethylphenol and 4-octanol, was prepared at a concentration of 50 ppm in methanol, separately. All the solutions were stored in dark bottles at −20 °C.

### 3.3. LiChrolut-EN-SPE and Solvent-Assisted Flavor Evaporation (SAFE)

The LiChrolut-EN cartridge was made by packing one-gram of resin into a 15 mL, 2 cm diameter reservoir (Thermo Scientific). The cartridge was conditioned with 10 mL of dichloromethane, air-dried, and then eluted with 10 mL of methanol, and finally washed with 10 mL of Milli-Q water. Two hundred and fifty milliliters of wine sample was percolated through the LiChrolut-EN under vacuum at 3 mL per min. The column was then washed with 10 mL of water and eluted with 20 mL of dichloromethane. The eluent was passed through the SAFE device (BÆNG; Glasbläserei Bahr, Manching, Germany) to remove the nonvolatile constituents at 50 °C under a vacuum of 2.80 × 10^−3^ torr. The distillate was concentrated in a Kuderna-Danish concentrator in 40 °C water bath with a Vigreaux column to approximately 5 mL. After drying over anhydrous sodium sulfate and transferring, the extract was further concentrated to 0.5 mL with a gentle stream of nitrogen.

### 3.4. GC-MS-Olfactometry Analysis

The GC-O analyses were performed on an Agilent 6890 GC (Agilent Technologies Inc., Santa Clara, CA, USA) equipped with an Agilent 5973 mass selective detector and a Gerstel Olfactory Detection Port (ODP). One microliter concentrated extract was injected in split mode (split ratio 1:10) and separated by a ZB-Wax column (30 m × 0.25 mm i.d., 0.50 μm film thickness, Phenomenex, Torrance, CA, USA). Helium was used as the carrier gas at a constant flow rate of 2.5 mL/min. At the exit of the capillary column, the effluents were split 1:1 (by volume) into a sniffing port and a MS detector. The GC injector and ODP temperature were both set at 250 °C. The oven temperature was programed at 40 °C for a 4 min holding and ramped up to 100 °C at a rate of 4 °C/min, then 3 °C/min to 230 °C with a 10 min holding. The MS transfer line and ion source temperature were 250 °C and 230 °C, respectively. Electron ionization mass spectrometric data from *m*/*z* 35–350 were collected using a scan rate of 5.27/s, with an ionization voltage of 70 eV. Three experienced panelists (two females and one male, with over 30 h of training) were selected for the GC-O analysis. The retention time, odor descriptor, and its intensity (5-point scale from 1 to 5 represent very weak, weak, moderate, strong, very strong) were recorded. Each sniffing session lasted 1 h and the panelists smelled each extract sample twice. The average intensity of the descriptors was calculated. A standard mixture of *n*-alkane C_5_–C_30_ was prepared and analyzed under the same GC conditions above. Retention Indices (RI) were calculated in accordance with a modified Kovats method based on individual retention times from the lab using pure reference compounds. Mass spectra of unknown compounds were compared with those present in the Wiley 275.L database (Agilent Technologies Inc.).

### 3.5. Quantitative Analysis of the Key Aroma-Active Compounds

#### 3.5.1. Static Headspace-GC-FID Analysis

Acetaldehyde, ethyl acetate, propanol, isobutyl alcohol, isoamyl acetate, and isoamyl alcohol were quantitated using the static headspace-GC-FID method described previously [46] due to their high concentrations in the sample. A Varian CP 3800 gas chromatograph equipped with a flame ionization detector (Varian, Inc., Palo Alto, CA, USA) was used. One milliliter of wine was added into a 20 mL auto sampler vial and 20 µL of internal standard (IS, 5 mg/L methyl propionate in methanol) was spiked. Samples were equilibrated at 70 °C for 15 minutes with agitation at 500 rpm. One thousand microliters of the headspace sample was injected using a heated (70 °C) gastight syringe (2.5 mL) in split mode 10:1. Separation was performed by a DB-FFAP capillary column (30 m × 0.32 mm i.d., 0.5 μm film thickness, Agilent Technologies). Helium was used as the carrier gas at a constant rate of 1.5 mL per minute. The oven temperature was set at 35 °C for 4 min holding, raised to 150 °C at a rate of 10 °C/min, and held at the final temperature for 5 min. The injector and detector temperature were both set at 250 °C, respectively. A standard calibration curve was prepared by spiking known amounts of standards into one mL of synthetic wine (12% ethanol (*v*/*v*), 3.5 g/L tartaric acid, pH 3.5) and 20 µL of IS (5 mg/L methyl propionate) was added. Data were collected by the Varian Star workstation. Standard curves and sample concentrations were calculated using interactive graphics.

#### 3.5.2. SBSE-GC-MS Analysis

For those compounds having low concentration or low affinity, the quantitation was conducted by the SBSE-GC-MS technique as described previously [47,48]. The comparison of two Twisters (PDMS and EG) is shown in the supplementary materials. A preconditioned PDMS coated stir bar (Twister) (10 mm × 0.5 mm, Gerstel Inc., Linthicum, MD, USA.) or an EG coated stir bar (0.5 mm film thickness, 10 mm length, Gerstel Inc.) was used to extract the aroma compounds. Ten milliliters of wine was pipetted into a 40 mL glass vial and diluted with 10 mL of saturated NaCl solution, and 20 µL of IS (50 ppm 4-octanol for PDMS stir bar set, 50 ppm 3,4-dimethlyphenol for EG stir bar set) solution was added. A PDMS or EG stir bar was then placed into the vial and stirred for 3 h at 1000 rpm at room temperature. After extraction, the stir bar was picked up from the vial, rinsed with Milli-Q water, dried with a Kimwipe, and transferred into a thermal desorption unit (TDU) for GC-MS analysis. Each sample was analyzed in triplicate. Analysis of the absorbed volatile compounds were performed on an Agilent 7890 GC coupled with a 5975 mass selective detector, and a Gerstel MPS-2 multipurpose TDU auto sampler with a CIS-4 cooling injection system (Gerstel Inc.). The analytes were thermally desorbed at the TDU in splitless mode. The CIS-4 was cooled to −80 °C with liquid nitrogen during the sample desorption, and then heated at 10 °C/s to 250 °C and held for 10 min for the PDMS bar, or to 220 °C for the EG bar. Solvent vent mode was used during the injection with a split vent flow of 50 mL/min. A ZB-WAX capillary column (30 m × 0.25 mm i.d., 0.5 μm film thickness, Phenomenex, Torrance, CA) was used. The oven temperature program was set at 40 °C for 4 min, raised to 230 °C at 4 °C/min, and held for 15 min. A constant helium flow of 2 mL/min was used. The MS transfer line and ion source temperatures were 280 °C and 230 °C, respectively. A standard calibration curve was prepared by spiking known amounts of standards into 10 mL of synthetic wine and 10 mL of saturated NaCl solution with 20 µL of IS. Each sample was analyzed in triplicate and the results were calculated through Chemstation software (v.10.1) (Agilent Technologies).

### 3.6. Sensory Evaluation of Wines and Aroma Reconstitution Test

The sensory evaluation of the two wine samples was conducted based on the method by Tao et al. [49]. The panel was trained over 50 days using a ‘‘Le Nez du Vin” aroma kit and 11 judges (4 males and 7 females) were selected. The Quantitative Descriptive Analysis (QDA) was performed to pick up the most important terms to describe the aroma characters of the wine samples in this study. The top (high *MF* values) 11 descriptors: fruity, berry, floral, black pepper, green or bell pepper, sour, caramel, smoky, woody, spicy, and rancid or cheesy were selected to describe the aroma of Syrah wine in this study. Sample wines in a balanced and completed block design were presented (in triplicate) to the panelists. They were required to use the 5 to 6 most significant terms as listed in Li et al. [37] to describe the wine aroma. Panelists were also asked to score the intensity of each term using a 5-point scale: (0) not detected; (1) weak, hardly recognizable note; (2) clear, but weak; (3) clear but not an intense note; and (4) intense note. The data processed were a mixture of intensity and frequency of detection (‘‘modified frequency”, *MF*), which was calculated with the formula proposed by Tao et al. [49,50]:

$$MF=\sqrt{F\left(\%\right)I\left(\%\right)}$$

*F* (%) is the detection frequency of an aromatic attribute expressed as a percentage; *I* (%) is the average intensity expressed as a percentage of the maximum intensity.

The aroma reconstitution test was conducted as described by Chen et al. [18] to resemble the wine according to the quantitative analysis and OAVs. Compounds with high OAVs (≥0.5) were reconstituted with the concentration in the matrix of synthetic wine (14% ethanol (*v*/*v*), 3.5 g/L tartaric acid, pH 3.5), labeled as RN and RY for NS and YS, respectively.

### 3.7. Statistical Analysis

The volatile compounds identified and quantified were listed in the table which was formed by Microsoft Office Excel 2013, and so was the mean value, standard deviation, and the OAVs. The concentration differences of volatiles between samples were determined using one-way analysis of variance (ANOVA) established by the Student’s t test at a significance level of ≤0.05, carried out using SPSS 20.0 (IBM, Armonk, NY, USA).

## 4. Conclusions

In this study, ethyl 2-methylpropanoate, ethyl 3-methylbutanoate, 3-methylbutyl acetate, 2- and 3-methyl-1-butanol, ethyl hexanoate, ethyl octanoate, 2-phenethyl acetate, methional, 3-methylbutanoic acid, hexanoic acid, octanoic acid, *β*-damascenone, guaiacol, 2-phenylethanol, *trans*-whiskylactone, 4-ethylguaiacol, eugenol, 4-ethylphenol, and sotolon were detected as the most significant aroma compounds in the GC-O analysis. β-Damascenone, ethyl octanoate, acetaldehyde, ethyl isovalerate, ethyl hexanoate, ethyl isobutyrate, isoamyl acetate, butanoic acid, 2- and 3-methylbutyric acid, hexanoic acid, and octanoic acid with high OAVs were detected as the most significant aroma contributors in the SBSE-GC-MS analysis. The candidate grape-derived compounds increased the understanding of Syrah wine produced in China. The fermentation-derived and oak-derived compounds identified in this study help winemakers and wine chemists better understand the aroma compositions and aroma profiles of the wine.

By comparing the odor intensities in GC-O, OAVs in the quantitative analysis, and scores of descriptors in the sensory evaluation, a more thorough understanding of the correlation of compounds and aroma was formed. Important aroma contributors: ethyl 2-methylbutyrate, ethyl hexanoate, ethyl octanoate, ethyl decanoate, 3-methylbutyl acetate, guaiacol, eugenol, and decanoic acid were detected at higher odor intensities in the sample Syrah extract from Helan Mountain as compared to Shangri-La. The quantitative analysis and OAV study also showed that most of the ethyl esters and acetate esters were found with higher OAVs in the Syrah wine of Helan Mountain rather than the wine from Shangri-La. It also should be noticeable that the Syrah wine from Ningxia in this study showed stronger fruity characteristics in the sensory evaluation study, which is consistent with the GC-O and quantitative analysis.

## Figures and Tables

**Table 1:** Odorants identified by Solid Phase Extraction-Gas Chromatography-Olfactometry (SPE-GC-O) in the two Syrah wines.

No.	Aroma Compounds ^a^	RI_ZB-wax_^b^	Odor Descriptions	Odor Intensity of ^c^	
				NS	YS
1	1,1-diethoxyethane	872	fruity, berry	1.5	2.0
2	ethyl acetate	895	fruity	2.5	2.3
3	ethyl propanoate	965	fruity	2.7	2.5
4	ethyl 2-methylpropanoate	974	fruity	3.7	3.5
5	2,3-butanedione	987	buttery	2.5	2.0
6	2-methylpropyl acetate	1018	fruity, lychee	1.9	2.3
7	ethyl butanoate	1041	dry fruit	2.8	2.7
8	butyl acetate	1056	fruity	2.8	2.7
9	ethyl 2-methylbutyrate	1060	fruity	2.7	2.3
10	2,3-pentanedione	1069	sour, fruity	2.0	2.3
11	ethyl 3-methylbutanoate	1074	fruity	3.5	3.5
12	2-methyl-propanol	1103	fusel	1.2	1.7
13	3-methylbutyl acetate	1132	fruity	3.7	2.5
14	ethyl pentanoate	1143	fruity	1.5	1.5
15	1-butanol	1163	whiskey, medicine	1.7	1.5
16	2- and 3-methyl-1-butanol	1223	medicine, brandy	5.0	5.0
17	ethyl hexanoate	1247	fruity	3.3	2.7
18	hexyl acetate	1268	fruity	1.5	1.3
19	3-hydroxy-2-butanone	1297	fruity	2.5	2.3
20	1-octen-3-one	1310	mushroom	1.2	1.5
21	3-methylpentanol	1335	fruity, green	1.5	1.3
22	ethyl lactate	1351	fruity	1.0	1.3
23	1-hexanol	1364	green	2.7	2.5
24	*cis*-3-hexenol	1401	green	1.7	1.5
25	*trans*-2-hexenol	1424	green	1.5	1.3
26	3-isopropyl-2-methoxypyrazine	1441	green pepper	1.7	1.5
27	ethyl octanoate	1444	fruity, floral	3.5	2.3
28	1-octen-3-ol	1461	mushroom	1.0	1.3
29	methional	1465	potato	3.5	3.0
30	furfural	1483	woody	1.5	1.7
31	3-*sec*-butyl-2-methoxypyrazine	1510	bell pepper	2.0	2.0
32	benzaldehyde	1525	almond	2.8	2.0
33	3-isobutyl-2-methoxypyrazine	1542	pepper	1.5	1.3
34	2-(methylthio) ethanol	1552	potato	2.2	2.3
35	linalool	1571	floral, citrus	1.2	1.5
36	ethyl 3-methylthiopropionate	1583	metallic, onion	1.7	1.3
37	2-methylpropanoic acid	1588	cheesy	1.5	2.0
38	butanoic acid	1628	sweaty	2.2	2.0
39	*γ*-butyrolactone	1642	nutty	1.2	1.0
40	ethyl decanoate	1651	fruity	2.2	1.7
41	furfuryl alcohol	1679	sweet, nutty	1.7	1.7
42	3-methylbutanoic acid	1687	sweaty	5.0	5.0
43	*α*-terpineol	1708	floral	2.7	3.2
44	3-(methylthio)propanol	1738	potato	2.2	2.0
45	*β*-citronellol	1759	floral	2.0	2.0
46	2-phenethyl acetate	1828	bread, sweet	2.7	3.0
47	*β*-damascenone	1841	tobacco, burnt sugar	4.2	4.0
48	hexanoic acid	1860	sweaty	2.8	3.0
49	geraniol	1865	citrus, floral	1.7	2.0
50	guaiacol	1872	phenolic, spicy	3.3	2.7
51	benzyl alcohol	1890	floral	2.8	2.7
52	*cis*-whisky lactone	1908	nutty, wood	3.5	3.8
53	2-phenylethanol	1935	floral, rose	4.5	4.2
54	*β*-ionone	1965	floral	1.7	1.5
55	*trans*-whisky lactone	1975	nutty, coconut	2.5	2.0
56	*trans*-2-hexenoic acid	1989	cheesy, herbal	1.7	1.7
57	*o*-cresol	2007	woody, phenolic	2.8	1.7
58	4-ethylguaiacol	2040	caramellic	3.3	3.2
59	*γ*-nonalactone	2049	nutty, woody	1.5	1.3
60	furaneol	2065	burnt sugar	3.5	3.2
61	octanoic acid	2078	sweaty	2.8	3.2
62	*p*-cresol	2087	horse	1.7	1.3
63	*m*-cresol	2103	leather	1.5	2.0
64	*γ*-decalactone	2149	fiber wood, sweet	2.5	1.5
65	ethyl cinnamate	2160	spice, sweet	2.5	2.0
66	eugenol	2179	honey, clove	3.5	2.7
67	4-ethylphenol	2194	medicine, horse	2.5	2.7
68	4-vinylguaiacol	2218	spice, anise	2.7	2.5
69	sotolon	2247	honey, caramel	3.3	2.7
70	2,6-dimethoxyphenol	2282	woody, phenolic	1.7	1.7
71	decanoic acid	2286	woody, rancid	2.7	1.3
72	ethyl anthranilate	2291	spice, sweet	2.7	1.7
73	isoeugenol	2366	sweet, floral	2.0	2.0
74	4-vinylphenol	2410	chemical, smoky	2.7	1.7
75	phenylacetic acid	2575	honey, rose	2.5	2.0
76	vanillin	2584	vanilla	2.7	1.7
77	methyl vanillate	2616	vanilla	1.8	2.0
78	ethyl vanillate	2658	vanilla	1.5	1.0
79	acetovanillone	2670	fruity, vanilla	1.7	1.3

^a^ Identification based on RI (compare retention index with authentic standards) and MS (mass spectrometry) or aroma description (A); ^b^ RI = retention index; ^c^ NS means Syrah wine from Ningxia Helan Mountain, YS from Yunnan Shangri-La.

**Table 2:** Volatile compounds quantified in two Syrah wine samples using Headspace GC-Flame Ionization Detector (HS-GC-FID) and Stir Bar Sorptive Extraction-GC-Mass Spectrometry (SBSE-GC-MS).

No.	Compounds	Odor *	Concentration ** (μg/L, mean ± SD)		OAV ***	
		Threshold	NS	YS	NS	YS
**Ethyl esters of straight-chain fatty acid**						
1	ethyl acetate	12,300	52,086 ± 3892 a	81,445 ± 5481 b	4.23	6.62
2	ethyl propionate	2100	204 ± 25	184 ± 47	<0.1	<0.1
3	ethyl butanoate	20	167 ± 12 a	120 ± 21 b	8.36	6.02
4	ethyl hexanoate	14	533 ± 32 a	151 ± 13 b	38.1	10.8
5	ethyl octanoate	5	275 ± 22 a	58 ± 8 b	54.9	11.7
6	ethyl decanoate	200	99.1 ± 5.3 a	16.3 ± 2 b	0.1–0.5	<0.1
**Ethyl esters of branched-chain fatty acid**						
7	ethyl isobutyrate	15	264 ± 24 a	164 ± 29 b	17.6	10.9
8	ethyl 2-methylbutanoate	18	79.6 ± 3.4 a	48.2 ± 5.4 b	4.42	2.68
9	ethyl isovalerate	3	131 ± 5 a	82 ± 9 b	43.8	27.3
**Higher alcohol acetates**						
10	isobutyl acetate	1800	78.1 ± 6.3 a	61.0 ± 10.1 b	<0.1	<0.1
11	butyl acetate	1600	23.4 ± 1.1 a	14.6 ± 1.6 b	<0.1	<0.1
12	isoamyl acetate	30	408 ± 26 a	244 ± 13 b	13.6	8.1
13	hexyl acetate	670	3.31 ± 0.37 a	0.76 ± 0.14 b	<0.1	<0.1
14	octyl acetate	50,000	3.85 ± 0.08 a	2.5 ± 0.12 b	<0.1	<0.1
**Aromatic esters and others**						
15	phenethyl acetate	73	33.3 ± 0.9 a	20.3 ± 1.2 b	0.1–0.5	0.1–0.5
16	ethyl phenylacetate	250	5.51 ± 0.26	5.92 ± 0.26	<0.1	<0.1
17	ethyl dihydrocinnamate	1.6	1.68 ± 0.58 a	0.41 ± 0.26 b	1.05	0.1–0.5
18	ethyl cinnamate	1.1	0.51 ± 0.13	0.37 ± 0.23	0.1–0.5	0.1–0.5
19	methyl anthranilate	3	0.7 ± 0.12 a	1.52 ± 0.24 b	0.1–0.5	0.5–1
20	ethyl anthranilate	16	0.39 ± 0.26 a	1.31 ± 0.26 b	<0.1	<0.1
21	methyl vanillate	3000	5.68 ± 2.62 a	13.5 ± 0.14 b	<0.1	<0.1
22	ethyl vanillate	990	499± 245	898 ± 307	0.5–1	0.5–1
23	diethyl succinate	120,000	11311 ± 1078	9736 ± 958	<0.1	<0.1
**Alcohols**						
24	1-propanol	50,000	31,180 ± 4642	38,252 ± 2941	0.5–1	0.5–1
25	isobutyl alcohol	40,000	52,872 ± 5530	47,188 ± 4978	1.32	1.18
26	isoamyl alcohol	30,000	123,435 ± 8642	105,594 ± 7374	4.11	3.52
27	1-hexanol	8000	1046 ± 35 a	1279 ± 63 b	0.1–0.5	0.1–0.5
28	*cis*-3-hexen-1-ol	1000	10.1 ± 4.9	12.7 ± 3.4	<0.1	<0.1
29	*trans*-2-hexen-1-ol	1000	6.98 ± 2.42	8.77 ± 3.16	<0.1	<0.1
30	1-octen-3-ol	20	14.3 ± 0.4 a	17.8 ± 0.9 b	0.5–1	0.5–1
31	benzyl alcohol	200,000	218 ± 52 a	595 ± 80 b	<0.1	<0.1
32	2-phenethanol	14,000	13793 ± 1724 a	9628 ± 801 b	0.5–1	0.5–1
**Fatty acids**						
33	butanoic acid	173	550 ± 63	484 ± 70	3.18	2.80
34	hexanoic acid	420	1489 ± 165 a	1076 ± 140 b	3.55	2.56
35	octanoic acid	500	650 ± 51	579 ± 68	1.30	1.16
36	decanoic acid	1000	122 ± 14 a	72 ± 16 b	0.1–0.5	<0.1
37	2-methylpropanoic acid	2300	730 ± 102 a	544 ± 96 b	0.1–0.5	0.1–0.5
38	2- and 3-methylbutyric acid	33	564 ± 134 a	390 ± 43 b	17.1	11.8
**Shikimic acid derivatives (volatile phenols)**						
39	guaiacol	23	28.8 ± 3.4 a	36.3 ± 3.1 b	1.25	1.58
40	4-methylguaiacol	65	1.98 ± 0.97 a	9.56 ± 0.15 b	<0.1	0.1–0.5
41	4-ethylguaiacol	33	1.23 ± 0.43 a	14.75 ± 0.69 b	<0.1	0.1–0.5
42	4-vinylguaiacol	1100	20 ± 4.5	17.8 ± 1.4	<0.1	<0.1
43	*o*-cresol	31	5.86 ± 2.2	3.19 ± 0.17	0.1–0.5	0.1–0.5
44	*p*-cresol	60	3.4 ± 1.64	4.44 ± 0.28	<0.1	<0.1
45	*m*-cresol	68	3.11 ± 1.69	2.36 ± 0.11	<0.1	<0.1
46	eugenol	6	6.71 ± 1.25	7.16 ± 3.19	1.12	1.19
47	isoeugenol	6	0.44 ± 0.23	0.61 ± 0.25	<0.1	0.1–0.5
48	4-ethylphenol	440	1.76 ± 0.61 a	101 ± 11 b	<0.1	0.1–0.5
49	3-ethylphenol	250	1.86 ± 0.12 a	87 ± 12 b	<0.1	0.1–0.5
50	4-vinylphenol	180	56.9 ± 13.3 a	81.2 ± 6.6 b	0.1–0.5	0.1–0.5
**Terpenoids**						
51	linalool	15	11.6 ± 0.3 a	1.81 ± 0.05 b	0.1–0.5	0.1–0.5
52	*α*-terpineol	250	14.6 ± 0.1 a	12 ± 0.7 b	<0.1	<0.1
53	citronellol	100	1.76 ± 0.03 a	0.66 ± 0.05 b	<0.1	<0.1
54	geraniol	30	6.13 ± 0.72 a	1.73 ± 0.36 b	0.1–0.5	<0.1
55	nerol	300	8.98 ± 0.65	10.4 ± 1.4	<0.1	<0.1
56	rose oxide	0.2	0.03 ± 0.01	0.04 ± 0.03	0.1–0.5	0.1–0.5
57	linalool oxide	3000	1.72 ± 0.36 a	5.69 ± 0.49 b	<0.1	<0.1
**C_13_-norisoprenoids**						
58	*β*-damascenone	0.05	2.81 ± 0.1 a	0.22 ± 0.03 b	56.1	24.4
59	*β*-ionone	5	0.12 ± 0.02	0.09 ± 0.08	<0.1	<0.1
**Ketone and lactones**						
60	*γ*-octalactone	400	6.24 ± 1.56	5.15 ± 0.6	<0.1	<0.1
61	*γ*-nonalactone	30	8.33 ± 0.86	11 ± 1.98	0.1–0.5	0.1–0.5
62	*γ*-decalactone	88	0.6 ± 0.04 a	1.34 ± 0.29 b	<0.1	<0.1
63	*γ*-undecalactone	150	5.61 ± 0.72 a	3.71 ± 0.83 b	<0.1	<0.1
64	*cis*-whiskylactone	74	12.7 ± 2.5 a	33.9 ± 1.3 b	0.1–0.5	0.1–0.5
65	*trans*-whiskylactone	32	31.5 ± 4.2	58.3 ± 2.1	0.5–1	1.82
66	2-aminoacetophenone	1.4	1.01 ± 0.15	1.18 ± 0.11	0.5–1	0.5–1
**Aldehydes**						
67	acetaldehyde	500	22,343 ± 1397 a	31,905 ± 2231 b	44.7	63.8
68	cinnamaldehyde	1180	0.18 ± 0.03 a	0.46 ± 0.16 b	<0.1	<0.1
69	vanillin	200	88.8 ± 6.7 a	110 ± 8 b	0.1–0.5	0.5–1
**Methoxypyrazines**						
70	3-isopropyl-2-methoxypyrazine	0.015	0.001 ± 0.0003	N.D.	<0.1	<0.1
71	3-*sec*-butyl-2-methoxypyrazine	0.015	N.D.	0.0012 ± 0.0004	<0.1	<0.1
72	3-isobutyl-2-methoxypyrazine	0.002	0.0017 ± 0.001	0.0012 ± 0.0004	0.5–1	0.5–1

* Odor threshold of the volatiles were presented in μg/L. They were measured in model wine, water/ethanol (90 + 10, *w*/*w*) unless indicated, and referenced from the literature: [3,34,35,37,38,39,40,41,42,43,44,45]; ** Different letters within rows indicates statistical differences by the Duncan test (*p* < 0.05); *** OAV means odor activity value, calculated as the ratio between the concentration of the individual compound in wine and the threshold concentration of this substance.

## Supplementary Materials

Supplementary File 1
