# Supplementary material for: Characterization of the Key Aroma Compounds in Chinese Syrah Wine by Gas Chromatography-Olfactometry-Mass Spectrometry and Aroma Reconstitution Studies
Source: Molecules. 2017 Jun 24;22(7):1045. doi: 10.3390/molecules22071045 (PMC6151980; doi:10.3390/molecules22071045)
Supplement: Supplementary File 1 [file molecules-22-01045-s001.docx]

1. Reagents and chemical standards

The volatile standard compounds below were provided by Sigma-Aldrich (St. Louis, MO, USA), TCI America (Portland, OR, USA), EKC Inc. (Rosemont, IL, USA), Alfa Aesar (Ward Hill, MA, USA), and EMD Chemical Inc. (Gibbstown, NJ, USA).

| Analyte | Purity(%) | Qualitative fragments (m/z) | Quantitative fragments (m/z) |
| --- | --- | --- | --- |
| *Esters* |  |  |  |
| ethyl acetate | 99.80% | N/A | N/A |
| ethyl propionate | 99.70% | 102 | 57 |
| ethyl butanoate | >98% | 88 | 71 |
| ethyl valerate | >98% | 88, 57 | 85 |
| ethyl hexanoate | >98% | 99 | 88 |
| ethyl octanoate | >98% | 101, 127 | 88 |
| ethyl decanoate | >98% | 101, 155 | 88 |
| ethyl 2-methylpropanoate | >98% | 116 | 71 |
| ethyl 2-methylbutyrate | >98% | 57, 85 | 102 |
| ethyl 3-methylbutyrate | >98% | 57 | 88 |
| ethyl phenylacetate | 99% | 91 | 164 |
| 2-methylpropyl acetate | 99.80% | 73 | 56 |
| butyl acetate | >98% | 73, 61 | 56 |
| 3-methylbutyl acetate | ≥97% | 87 | 70 |
| hexyl acetate | >98% | 61, 69 | 56 |
| octyl acetate | >98% | 84, 70 | 83 |
| phenethyl aceate | 99% | 91 | 104 |
| diethyl succinate | >98% | 101 | 129 |
| ethyl cinnamate | >98% | 103 | 131 |
| ethyl hydrocinnamate | 99% | 91 | 104 |
| methyl anthranilate | 99% | 151 | 119 |
| ethyl anthranilate | >98% | 119 | 165 |
| methyl vanillate | 99% | 182 | 151 |
| ethyl vanillate | 99% | 151 | 196 |
| *Alcohols* |  |  |  |
| 1-propanol | 99.50% | N/A | N/A |
| 2-methyl-propanol | 99.50% | 43 | N/A |
| 2/3-methyl-1-butanol | >98% | 55, 70 | N/A |
| 1-hexanol | 99% | 69 | 56 |
| Z-3-hexan-1-ol | 90% | 82, 55 | 67 |
| E-2-hexen-1-ol | 96% | 82 | 57 |
| benzyl alcohol | 99.80% | 108 | 79 |
| 2-phenylethanol | 99% | 92, 122 | 91 |
| 1-octen-3-ol | 96% | 57 | 72 |
| 4-octanol | ≥97% | 69, 73, 87 | 87 |
| *Fatty Acids* |  |  |  |
| propanoic acid | 99% | 45, 57 | 74 |
| 2-methylpropanoic acid | 99% | 88 | 73 |
| butanoic acid | 99% | 73 | 60 |
| 2-methylbutanoic acid | 99% | 57 | 74 |
| 3-methylbutanoic acid | 99% | 57 | 74 |
| hexanoic acid | ≥98.0% | 73, 87 | 60 |
| octanoic acid | ≥98.0% | 73, 85 | 60 |
| decanoic acid | 99% | 73 | 129 |
| *Volatile Phenolics* |  |  |  |
| o-cresol | 99% | 79, 90 | 108 |
| m-cresol | 99% | 107, 77 | 108 |
| p-cresol | 99% | 108, 77 | 107 |
| guaiacol | ≥98.0% | 81, 124 | 109 |
| 4-methylguaiacol | 99% | 138, 95 | 123 |
| 4-ethylguaiacol | ≥98.0% | 152, 122 | 137 |
| 4-vinylguaiacol | 97% with 0.01% BHT | 135 | 150 |
| eugenol | 98% | 149 | 164 |
| isoeugenol | 98% | 149 | 164 |
| 4-ethylphenol | 98% | 122 | 107 |
| 3-ethylphenol | 95% | 122, 77 | 107 |
| 4-vinylphenol | 98% | 91 | 120 |
| *Terpenoids and C13-norisprenoids* | |  |  |
| linalool | 97% | 93 | 121 |
| α-terpineol | ≥96% | 121 | 136 |
| β-citronellol | 95% | 95, 69 | 81 |
| nerol | 98% | 41, 93 | 69 |
| geraniol | 98% | 69,123 | 93 |
| rose oxide | mixture of isomers, ≥97.0% | 69 | 139 |
| linalool oxide | mixture of isomers, ≥97.0% | 111 | 93 |
| β-damascenone | ≥98% | 69 | 121 |
| β-ionone | 95% | 135 | 177 |
| *Lactones and Ketones* |  |  |  |
| γ-octalactone | 98% | 114, 57 | 85 |
| γ-nonalactone | 98% | 57, 100 | 85 |
| γ-decalactone | 98% | 55 | 85 |
| γ-undecalactone | 98% | 128 | 85 |
| E-whiskeylactone | 98% | 69, 71 | 99 |
| 1-octen-3-one | ≥97.0% | 55 | 83 |
| 2-aminoacetophenone | 95% | 135 | 120 |
| *Pyrazines* |  |  |  |
| 3-isopropyl-2-methoxypyrazine | 99% | 137 | 124 |
| 2-secbutyl-3-methoxypyrazine | 99% | 138, 151 | 124 |
| 3-isobutyl-2-methoxypyrazine | 99% | 94 | 124 |
| *Aldehyde* |  |  |  |
| acetaldehyde | ≥99% | N/A | N/A |
| cinnamaldhyde | ≥98% | 103 | 131 |
| vanillin | 98% | 81 | 151 |

1. Comparison of PDMS and EG stir bar in the extraction of volatile compounds

SBSE-GC/MS methods for the determination of volatile compounds in the Syrah samples using two types of Twister were comparatively studied. Based on the response of each compounds in the GC-MS analysis, their average of the triplicate peak areas was logarithm calculated as the “y” axis, with seven or nine concentration levels as the “x” axis. The responses of the internal standard 4-octanol and 3, 4-dimethylphenol in 9 levels were also calculated, shown in Figure 1e. As shown in Figure 1a–d (only the high contributing compounds were shown here), SBSE using PDMS Twister and EG-Silicone/PDMS Twister gave similar results in ethyl branched esters, acetate esters, fusel alcohols, terpenols, and lactones compound groups. However, PDMS Twister showed better extraction efficiency in ethyl straight esters and β-damascenone compounds than EG did. In contrast, EG Twister presented greater extraction capacity in 2-phenethanol, straight and branched fatty acids, and phenols (except eugenol). Whiskylactone was shown to have a special issue: PDMS was better in the low concentration range than EG. Vanillin derivatives could not even be extracted by PDMS until 50 μg/L. As shown in Figure 1e, EG-Silicone twister obtained a significantly higher response of 3,4-dimethylphenol. According to the manual (Gerstel Inc. U.S.A.), the PDMS Twister’s maximum desorption temperature can be up to 300 °C, which enables better and more complete conditioning and smaller background noise, and overall the PDMS Twister offers better long-term stability than the EG-Silicone Twister; EG-Silicone Twister allows extraction from wine samples without derivatizations, saving time and resources. In this study, only phenols were calculated by the EG Twister extraction in the following quantitative analysis. The rest of the quantified compounds were all extracted by PDMS Twister.

**Figure 1.** Comparison of the response of PDMS and EG Twister on the typical compound standards.


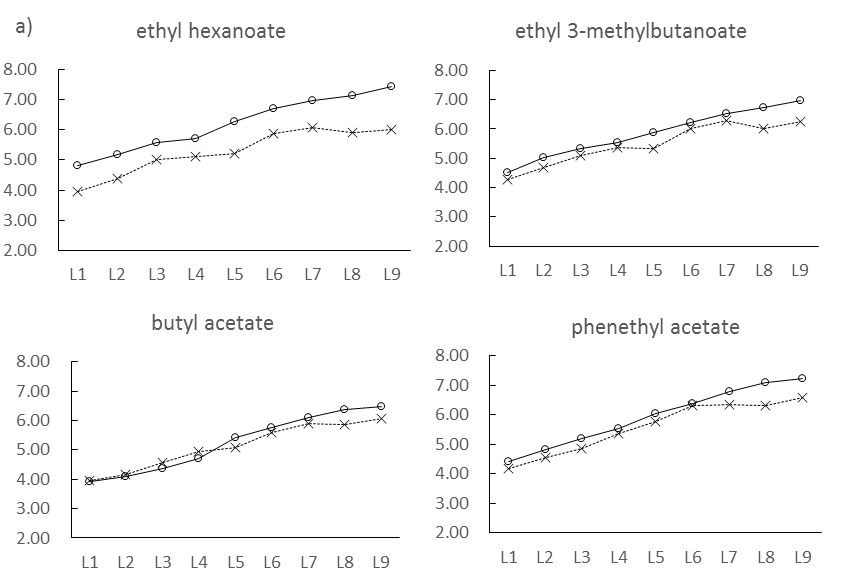


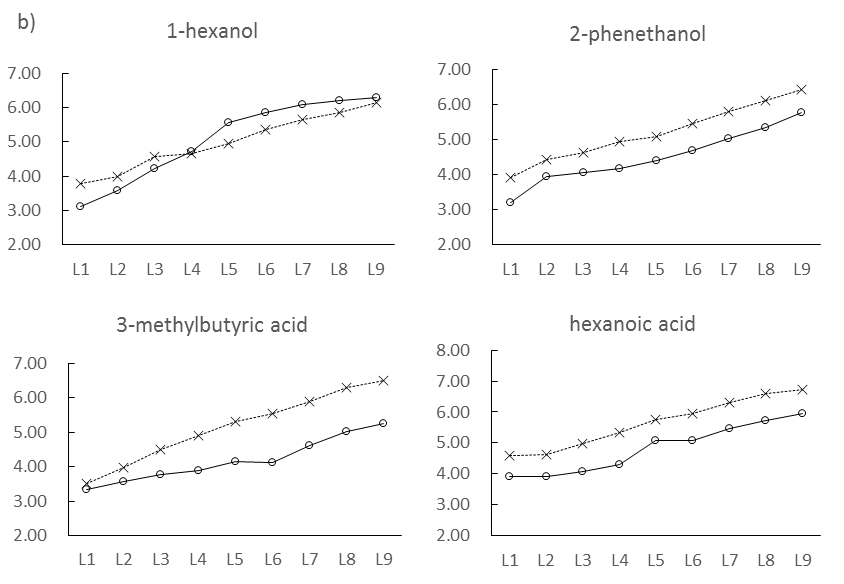


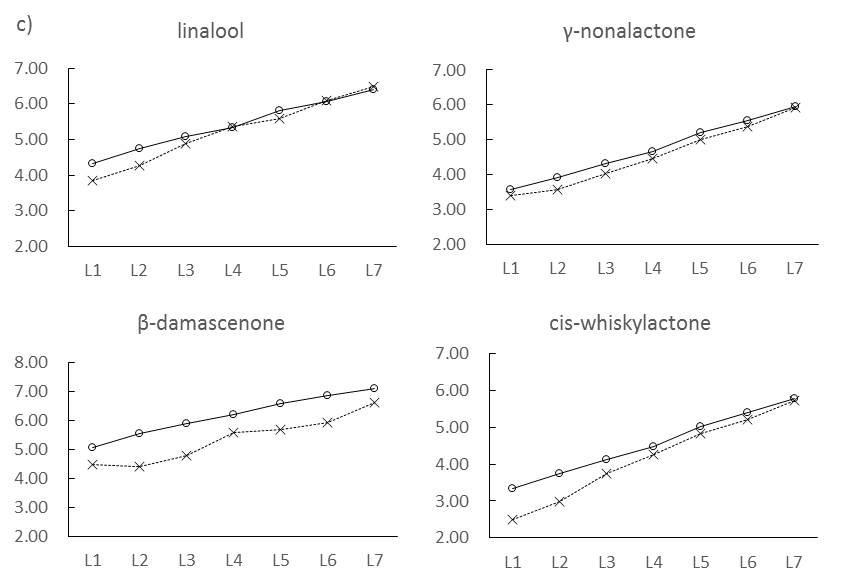


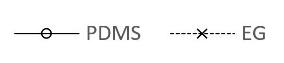

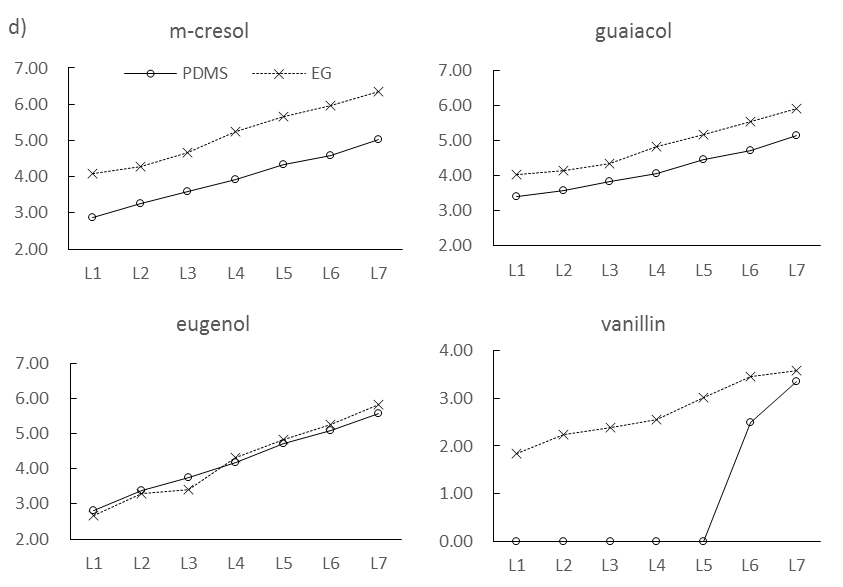


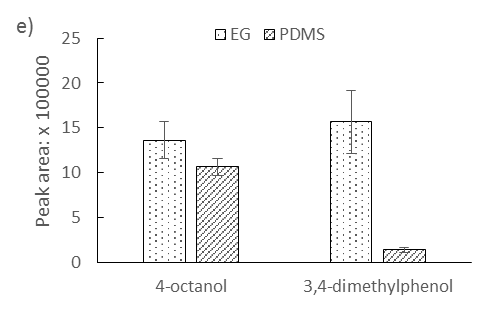


| Symbols | L1 | L2 | L3 | L4 | L5 | L6 | L7 | L8 | L9 |
| --- | --- | --- | --- | --- | --- | --- | --- | --- | --- |
| Concentration of standards (μg/L) | 1.0 | 2.5 | 5.0 | 10 | 25 | 50 | 100 | 250 | 500 |

^a^ In Figure 1a–d, the “y” axis was logarithm calculated from the peak area of the compounds extracted by the two stir bars. In Figure 1e, the “y” axis is the mean values of the peak areas of the internal standards detected by GC-MS.
